# Correction: A clinical and computational study on anti-obesity effects of hydroxycitric acid

**DOI:** 10.1039/c9ra90051a

**Published:** 2019-07-18

**Authors:** Manu Tomar, Raghavendra Pralhada Rao, Palaniyamma Dorairaj, Abhishek Koshta, Sowbhagya Suresh, Mohamed Rafiq, Rajesh Kumawat, Rangesh Paramesh, Babu U. V, K. V. Venkatesh

**Affiliations:** Department of Chemical Engineering, Indian Institute of Technology, Bombay Powai Mumbai – 400076 India venks@iitb.ac.in +91 22 2576 7223; The Himalaya Drug Company Makali Bengaluru 562162 India dr.babu@himalayawellness.com +91 8067549862

## Abstract

Correction for ‘A clinical and computational study on anti-obesity effects of hydroxycitric acid’ by Manu Tomar *et al.*, *RSC Adv.*, 2019, **9**, 18578–18588.

The authors regret that the name of one of the authors (Raghavendra Pralhada Rao) was shown incorrectly in the original article. The corrected author list is as shown above.

The Royal Society of Chemistry apologises for these errors and any consequent inconvenience to authors and readers.

## Supplementary Material

